# Empowering Heart Failure Patients: The Role, Importance, and Considerations of Active Patient Engagement in Improving Delivery of Guideline-Directed Medical Therapy

**DOI:** 10.1177/23743735251380970

**Published:** 2025-09-24

**Authors:** Michele Senni, Beth Towery Davidson, Naresh Kanumilli, Teresa Levitch, Corrado Farchioni, Cynthia Chauhan, Petrina Stevens, Elmas Malvolti, Ana Marija Gjurovic, Caroline Brigitte Katzer, James King, Javed Butler

**Affiliations:** 1 Cardiology Unit, Cardiovascular Department, 9333 Hospital Papa Giovanni XXIII, Bergamo, University of Milano, Bicocca, Italy; 2 19152 Abbott Laboratories, Pleasanton, CA, USA; 3 434280 Northenden Group Practice, Manchester, UK; 4 Patient Author, Poughkeepsie, NY, USA; 5 Patient Author, Villasanta, Italy; 6 Patient Author, Wichita, KA, USA; 7 Global Medical Evidence, BioPharmaceuticals Business Unit, AstraZeneca, Cambridge, UK; 8 Global Medical Affairs, BioPharmaceuticals Business Unit, AstraZeneca, Cambridge, UK; 9 427918 OPEN Health, London, UK; 10 466551 Baylor Scott and White Research Institute, Dallas, TX, USA; 11 University of Mississippi Medical Center, Jackson, MS, USA

**Keywords:** heart failure, patient empowerment, patient education, behavioral science, shared decision making

## Abstract

Heart failure (HF) is a chronic syndrome that is associated with frequent rehospitalization. Patient-focused research suggests that increased health-management activation may lead to favorable clinical outcomes. This article introduces a case study of the development of a patient education and activation tool for use by HF-specialist clinicians, “ENGAGE in HF,” and advocates for wider adoption of co-creation involving people living with HF. Following a behavioral assessment of unmet needs in the HF-patient population in scientific literature, people living with HF were invited to comment on the content plan and excerpts from the tool. Afterwards, HF-specialist clinicians provided their scientific perspective and assessed the content's practical utility. The co-created tool aims to improve dialogue between clinicians and patients. It allows clinicians to personalize conversations, considering each patient's capacity to integrate information and their readiness to engage in shared decision making. Feedback from initial dissemination of the ENGAGE in HF tool among cardiologists in China suggests that use of such tools may lead to improvements in patient education, symptom management, and adherence to medication.

## Summary Points

- Heart failure (HF) is a long-term health problem linked with frequent rehospitalization.
- Behavioral science can help us to support people with HF in feeling ready to make positive lifestyle changes.
- ENGAGE in HF is a new tool helping clinicians provide personalized education for people with HF. It was made with the help of people with HF and clinicians who treat HF.
- By co-creating behavior-focused support materials with people living with HF and clinicians, we hope to better meet healthcare service users’ needs, increase the use of these tools in everyday practice, and improve clinical outcomes.

## Introduction to the Issue

### Background

Up to 64 million people (around 1%-2% of the adult population) are currently living with heart failure (HF), a life-limiting condition.^1–3^ HF is a chronic syndrome linked with many comorbidities, particularly diabetes, ischemic heart disease, obesity, cancer, and chronic kidney disease.^2,4^ Consequently, HF is implicated in 1%-2% of all hospital admissions and the average person with HF is hospitalized approximately once per year.^1^

### Key Challenges for Clinicians

Many clinicians have limited time for individual appointments, which reduces their ability to take a personalized approach to the patient in front of them. The consequence is a “one-size-fits-all” care strategy that doesn’t consider individual needs. Clinicians under pressure may also be reluctant to shift from established but outdated treatment strategies.^5^

### Key Challenges for People With HF

Limited healthcare resources have impacts on patients too. People with HF often have limited support to be empowered to self-manage their condition, increasing their risk of hospitalization.^5^ When patients feel alienated from decisions about their care, they may not understand the need for adherence to treatment or may fail to report side effects that impact their quality of life.^5^ This may, at least partially, explain why people with HF often have a residual risk with poor outcomes, even when they have been prescribed guideline-directed medical therapy (GDMT).^5^

### The Unmet Need in HF

Clinicians and people with HF need to be supported to implement GDMT in practice. Better educated and empowered patients – and caregivers – can take an active role in their care, maintain high adherence to clinician recommendations and therapy, and avoid hospitalizations.^6^

When patients participate more actively and knowledgeably in their care, healthcare providers may find it easier to overcome clinical inertia and implement GDMT earlier.^5^

The aim is that those improvements in clinical practice can lead to fewer hospitalizations and more efficient use of healthcare resources, as well as better outcomes overall.^5^

### Why is Behavioral Science Important?

People with HF have behavioral traits and external factors that can act as barriers and/or enablers to making changes in their lives and influence their level of health empowerment. Research into the drivers and barriers to human behavior change categorizes such factors into those affecting capability, opportunity, and motivation.^7^ Understanding the factors that drive patient motivation and ability to engage in their own care may support the implementation of personalized behavior change techniques through novel interventions.^8^ Ultimately, developing more personalized solutions to healthcare engagement may help clinicians provide better support that goes beyond education, by addressing behavioral barriers and improving communication in the healthcare setting.

Through such an approach, we aim to change the patient narrative from the passive experience of *receiving* care to actively participating in and being responsible for one's own care. These interventions should permeate care, beginning from diagnosis, so that people living with HF form an accurate knowledge base, with a goal to make positive health changes.

### Limitations to Existing Interventions

To date, clinicians use several strategies to improve self-management among people with HF^6^:

- Face-to-face education;
- Weekly phone call follow ups;
- Group training sessions;
- eLearning programs; and
- Other non-eLearning programs (eg, educational handbooks, brochures, etc).

These interventions may improve self-care behaviors and quality of life, but they carry significant limitations:

- Requiring too much clinician/patient time for training and delivery;
- Being too expensive for already burdened healthcare services; and
- Taking a “one-size-fits all” approach by necessity, rather than considering personal barriers, behavioral traits, and learning preferences.

Increasing the use of behavioral science to understand patients may help drive active participation by patients and clinicians to accelerate patient empowerment.

## Description of the Intervention

### Introducing ENGAGE in HF

**Figure 1.**
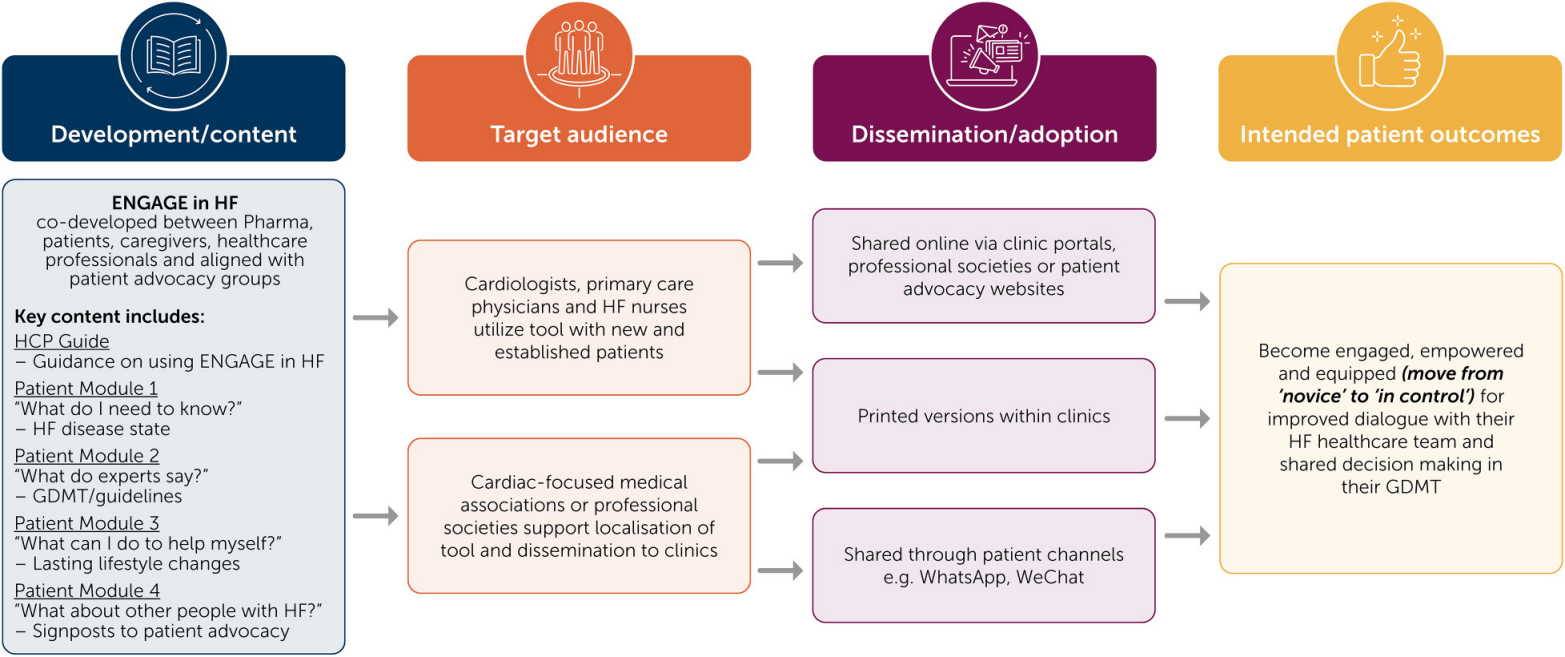
Implementation plan for the ENGAGE in HF tool.

Abbreviations: GDMT, guideline-directed medical therapy; HCP, healthcare professional; HF, heart failure.

ENGAGE in HF is an educational tool designed to facilitate improved communication between clinicians and people with HF (Figure 1). It applies the principles of behavioral psychology by addressing the barriers and enablers to self-management, proactive care engagement and shared decision making. ENGAGE in HF aims to improve satisfaction with care and care provision, as well as health outcomes for those living with HF, including:

- Early, proactive recognition and action on worsening signs of HF;
- Improving the ease of personalized care provision for healthcare providers;
- Improving adherence to prescribed care plans; and
- Encouraging better collaboration between patients and clinicians.

The ENGAGE in HF tool contains distinct modules that focus on specific aspects of the HF journey:

1. Education on the basic biology behind HF, how it is diagnosed and classified, and how to respond to worsening symptoms;
2. Information about how HF is monitored, test results, and how treatments work;
3. Guidance on making tangible, sustainable lifestyle changes to improve quality of life; and
4. Advice on talking about HF with loved ones, and signposting to support in the wider HF community such as international patient networks.

### What Makes ENGAGE in HF Different?

#### Incorporating Behavioral Science

ENGAGE in HF was developed from research into effective behavior change, beginning with identifying modifiable behavioral traits through a small, targeted review of published literature. Our research indicated that people with heart failure generally have low health literacy, which limits their capability, opportunities, and motivation to engage in effective self-care or take part in decisions about treatment.^9^

These traits were then used to map patients on a health empowerment continuum, describing them as either a “*Novice*,” “*In the know*,” or “*In control*” to help us target educational modules and behavioral techniques to those who would benefit most. The ultimate aim was to facilitate people with HF's advancement along this continuum to become empowered self-advocates.

Within the tool, the continuum was used to create a rapid decision tree to help clinicians and patients choose the right content at the right time in a person's health journey.

In module 1 “What do I need to know?” and module 2 “What do experts in HF say?”, the “*Novices*” learn the basics of their condition and practical behaviors for self-management, raising their capabilities and opportunities to engage in self-care and shared decision making. As an example, after describing how to identify acutely worsening HF (eg, unexplained weight gain, shortness of breath, and chest pain), we include an action plan template that patients can develop with their healthcare team for use in an emergency. Information about their disease is structured according to the domains defined in Leventhal's Common Sense Model.^10^

Those who are “*In the know*” have a foundation of understanding but need support putting their knowledge into practice (eg, adherence to medication and lifestyle changes). For this group, module 3 “What can I do to help myself?” combines empathetic discussion of the challenges of life with HF with motivational content. Here, we focus on the importance of setting small, achievable goals for lasting change.

Finally, those who are “*In control*” of their condition are ready to become advocates for themselves and for other people with HF. In the final module of the tool “What about other people with HF?”, we provide advice on having conversations about HF with friends and family, including practical help that individuals can request for shopping and household chores. We also include signposts to patient advocacy networks such as the World Heart Federation while recommending readers try to find local patient groups with the help of their healthcare team.

#### Co-Creating With Stakeholders

ENGAGE in HF was co-created with people with HF and specialist clinicians to ensure that the content was relevant, useful and accessible.

Eleven people with HF provided their feedback through an online workshop platform, guided by questions about the content, as well as commenting directly on the materials. Changes made because of reviewer feedback included more use of colloquially understood terms like “echo” in place of “transthoracic echocardiography,” more use of illustrations and diagrams in place of dense text, and greater emphasis on positive, actionable language.

Three clinicians examined later drafts of the modules and provided their expertise on scientific accuracy and relevance to clinical practice. A key intervention was their support in developing the aforementioned emergency action plan for people experiencing acute worsening of HF symptoms. They also provided recommendations for describing medical devices to manage heart failure, such as cardiac resynchronization therapy pacemakers, using accessible language used in their clinics.

#### Dissemination

The first dissemination of ENGAGE in HF took place in January 2024 in China through 2 main channels:

1. As a downloadable item shared on the Chinese Cardiovascular Association “Heart Failure Center” website and WeChat social media app and
2. As printed booklets distributed to 21 specialist heart failure centers.

**Figure 2.**
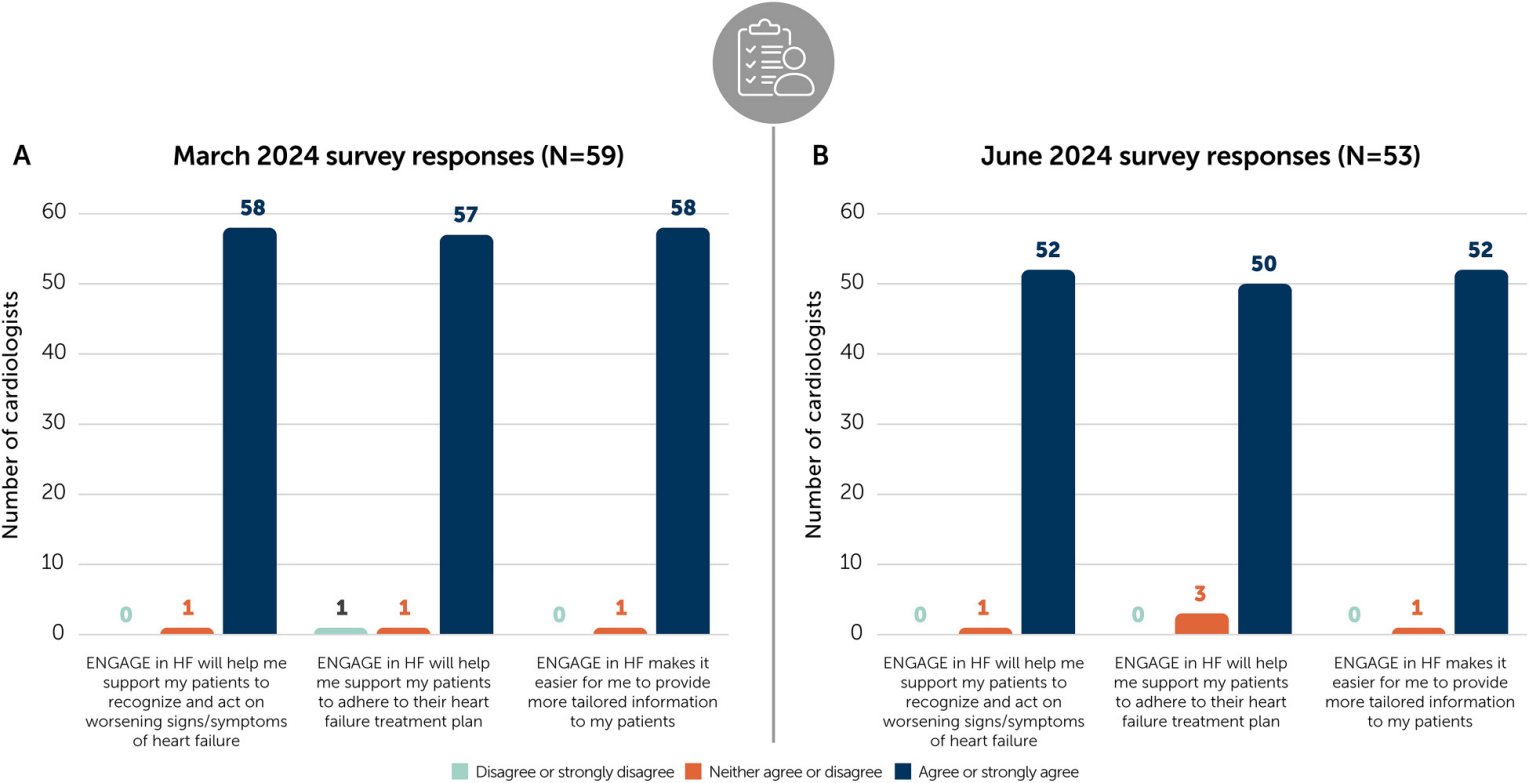
Results of surveys sent to cardiologists at 21 centers in China in March (A) and June (B) 2024. HF, heart failure.

AstraZeneca conducted surveys of cardiologists from those centers in March and June 2024. Of 59 cardiologists surveyed in March 2024, 58 felt confident that the tool would help their patients to recognize and act on worsening signs of HF. Furthermore, 58 healthcare professionals felt that ENGAGE in HF made it easier for them to provide tailored information to their patients (Figure 2A). In the June follow-up survey, 50 out of 53 felt the tool would help to support patients in adhering to their treatment plan (Figure 2B).

## Conclusion

To improve patient outcomes, a fundamental change is required in how to approach and empower patients across healthcare services. Clinicians need to recognize patients and caregivers as critical members of the team and include them in shared decision making.

We believe that clinicians may benefit from tools and skills that help people with HF to actively participate in their own care. An essential step to achieve this is by identifying an individual's unique needs, motivations, and barriers to change, and then tailoring health interventions accordingly.

The development of such tools may be supported by partners, such as hospital systems, insurance companies, and the pharmaceutical industry. Then with support from local experts, the content may be further refined for specific cultural and regulatory contexts.

Our hope for the future of clinical practice is that greater patient empowerment will lead to improved use of GDMT, more regular follow ups, less frequent hospitalizations and, ultimately, optimized patient outcomes.
